# Vascular protective effect of aspirin and rivaroxaban upon endothelial denudation of the mouse carotid artery

**DOI:** 10.1038/s41598-020-76377-8

**Published:** 2020-11-09

**Authors:** T. G. Mastenbroek, M. F. A. Karel, M. Nagy, W. Chayoua, E. I. J. Korsten, D. M. Coenen, J. Debets, J. Konings, A. E. Brouns, P. J. A. Leenders, H. van Essen, R. van Oerle, S. Heitmeier, H. M. Spronk, M. J. E. Kuijpers, J. M. E. M. Cosemans

**Affiliations:** 1 Department of Biochemistry, Cardiovascular Research Institute Maastricht (CARIM), Maastricht University, Maastricht, The Netherlands; 2 Synapse Research Institute, Cardiovascular Research Institute Maastricht (CARIM), Maastricht University, Maastricht, The Netherlands; 3 Department of Pharmacology & Toxicology, Cardiovascular Research Institute Maastricht (CARIM), Maastricht University, Maastricht, The Netherlands; 4 Department of Complex Tissue Regeneration, MERLN Institute for Technology-Inspired Regenerative Medicine, Maastricht, The Netherlands; 5 Cardiovascular Research Institute, Bayer AG, Wuppertal, Germany

**Keywords:** Animal disease models, Platelets, Cardiovascular diseases

## Abstract

While in recent trials the dual pathway inhibition with aspirin plus rivaroxaban has shown to be efficacious in patients with atherosclerotic cardiovascular disease, little is known about the effects of this combination treatment on thrombus formation and vascular remodelling upon vascular damage. The aim of this study was to examine the effects of aspirin and/or rivaroxaban on injury-induced murine arterial thrombus formation in vivo and in vitro, vessel-wall remodelling, and platelet-leukocyte aggregates. Temporary ligation of the carotid artery of C57BL/6 mice, fed a western type diet, led to endothelial denudation and sub-occlusive thrombus formation. At the site of ligation, the vessel wall stiffened and the intima-media thickened. Aspirin treatment antagonized vascular stiffening and rivaroxaban treatment led to a positive trend towards reduced stiffening. Local intima-media thickening was antagonized by both aspirin or rivaroxaban treatment. Platelet-leukocyte aggregates and the number of platelets per leukocyte were reduced in aspirin and/or rivaroxaban treatment groups. Furthermore, rivaroxaban restricted thrombus growth and height in vitro. In sum, this study shows vascular protective effects of aspirin and rivaroxaban, upon vascular injury of the mouse artery.

## Introduction

Atherosclerosis, i.e. artery lumen narrowing due to plaque formation, is the main pathology underlying both coronary artery disease (CAD) and peripheral artery disease (PAD). Rupture or erosion of atherosclerotic plaque simultaneously triggers platelet activation and activation of the extrinsic coagulation cascade. Platelets steer coagulation in several ways, e.g. by expressing a procoagulant surface to which vitamin-K dependent coagulation factors can bind to via calcium, by releasing several (anti)coagulation factors, by providing a scaffold for the formation of fibrin fibers and by promoting clot retraction^1^. Vice versa, thrombin, a key enzyme in the coagulation cascade, potently activates platelets via protease-activated receptors (PARs)^2^. Next to their vital role in atherothrombosis, platelets and coagulation also promote atherosclerosis initiation and progression, by exerting pro-inflammatory activities^3–5^. In the clinic, Dual Pathway Inhibition (DPI) with the platelet inhibitor aspirin and low dose Factor Xa (FXa) inhibitor rivaroxaban has been demonstrated to lead to reduced major cardiovascular events in patients with stable CAD or PAD, when compared to monotherapy with aspirin or rivaroxaban^6,7^. Discussion points concerning the implementation of DPI concern defining the patient groups that will benefit most from DPI, defining the optimal rivaroxaban concentration and the duration of the treatment^8–10^. The current knowledge on the molecular rationale and clinical evidence for DPI is expertly reviewed by Weitz et al.^9^ and Gurbel et al.^11^. In particular the vascular-directed effects of platelets and coagulation factors are not fully known at present. What it is known it that for the coagulation cascade, atherogenic effects have in particular been reported for FXa and thrombin, via interaction with PARs expressed on a range of vascular cells^4^. Platelets promote atherosclerosis by release of cytokines and growth factors and, importantly, by promoting leukocyte recruitment and extravasation to inflammatory sites^12^. Platelet-leukocyte aggregates (PLAs) are considered to be a marker for cardiovascular disease^13^. PLA formation in the blood or at the site of activated or damaged endothelial cells involves several receptor-ligand pairs, of which the interaction of platelet P-selectin, which is expressed upon platelet activation, with leukocyte P-selectin glycoprotein ligand-1 (PSGL-1) is considered as an important initial step^14^. Major agonists involved in PLA formation are adenosine diphosphate (ADP) (e.g. released by platelets and red blood cells) and thrombin (generated by the coagulation system)^15^. Interestingly, in patients with cardiovascular disease, P2Y_12_ inhibition potently reduced PLA formation, whereas aspirin is less potent or potentially even without effect^16,17^. To our knowledge, no studies have been published on the effect of rivaroxaban on PLA formation.

Platelet activation has also been associated with vascular stiffening in healthy volunteers^18^ and in subjects with diabetes, hypertension and hyperlipidaemia^19^. Vascular stiffening of the arteries is a result of pathological arterial remodelling. It is considered to have a negative impact on the heart and vasculature, and is associated with an increased risk for cardiovascular disease^20^. However, the mechanisms by which activated platelets may contribute to vascular stiffening are still unknown. Moreover, studies investigating the effect of aspirin or rivaroxaban treatment on arterial stiffness show either conflicting results, as is the case for aspirin^21–23^, or are scarce, as is the case for rivaroxaban^24^.

The aim of this study is to assess the effects of treatment with aspirin and/or rivaroxaban on injury-induced murine arterial thrombus formation and vessel-wall remodelling.

## Results

### Temporary ligation of the mouse carotid artery leads to endothelial denudation and sub-occlusive thrombus formation persisting for several days

**Figure 1:**
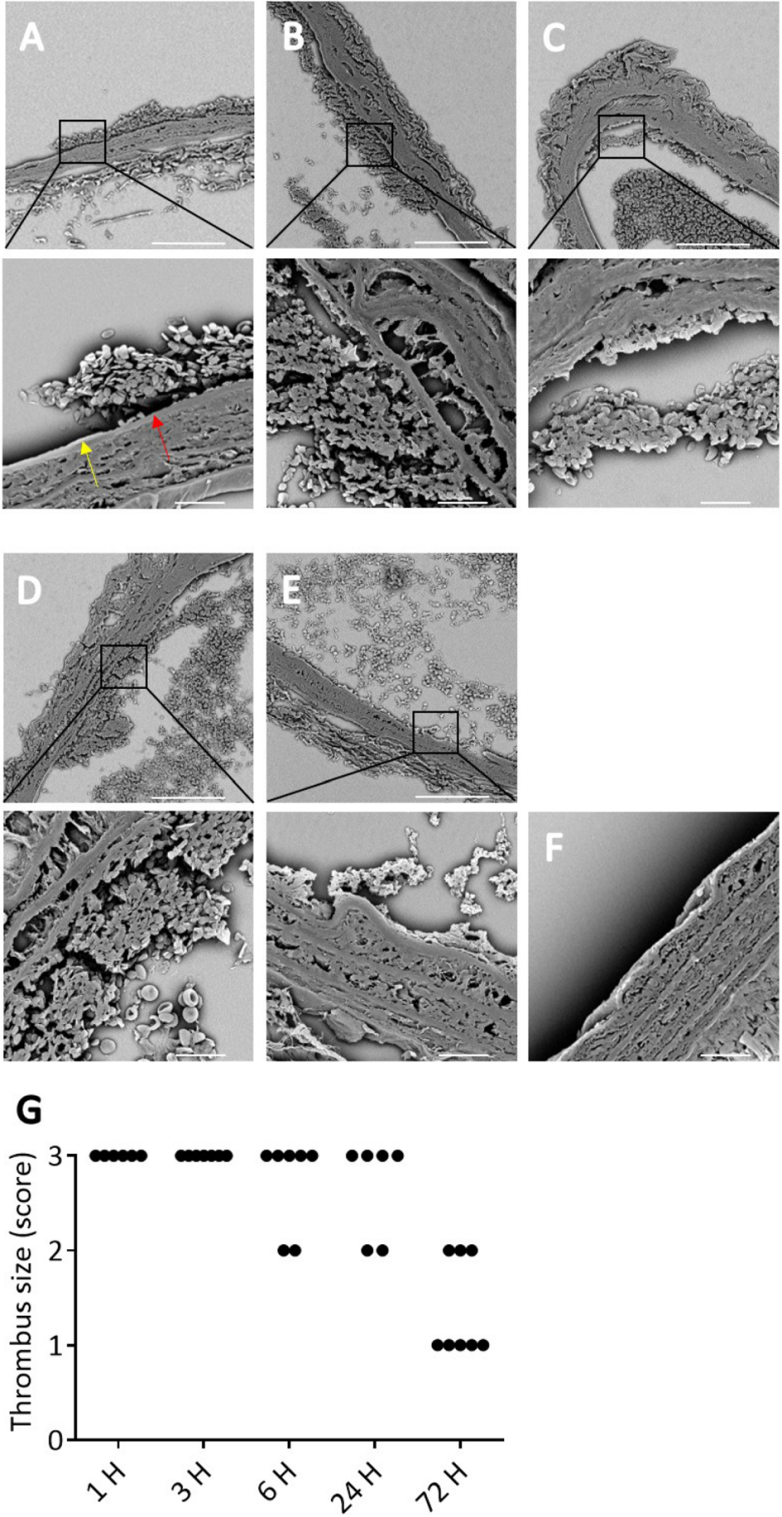
Temporary ligation of the carotid artery provokes consistent sub-occlusive thrombus formation in vivo. Scanning electron microscopy (SEM) images of thrombi (**A**) 1, (**B**) 3, (**C**) 6, (**D**) 24 and, (**E**) 72 h after temporary ligation of the carotid artery in control WT mice. (**A–E**) In all samples sub-occlusive platelet-rich thrombus formation is observed on the injured vessel wall. Damaged endothelium is indicated by a red arrow and undamaged endothelium by a yellow arrow. (**F**) No platelet adhesion was observed in absence of temporary ligation (3 mm upstream). Representative SEM images of 6 independent ligations are depicted. Scale bar is 80 μm, or 10 μm for zoomed images. (**G**) Thrombus size was scored by examining SEM images obtained from ligated carotid arteries that were collected at 1, 3, 6, 24 or 72 h after ligation. Scoring scale: 0 = no platelet adhesion, 1 = platelet monolayer, 2 = small thrombi (< 15 layers), and 3 = larger thrombi (> 15 layers).

In this study an experimental mouse model was developed to characterise the effect of aspirin and/or rivaroxaban on vascular remodelling upon vascular damage. Vascular injury was inflicted by a temporary ligation of the common carotid artery of C57BL/6 mice, two times for a duration of 60 s each. Model characterisation was done by determining the size of formed thrombi by intravital and electron microscopy and the extent of vascular damage by histology. Temporary ligation (2 times 1 min) of the carotid artery led to platelet adhesion and sub-occlusive thrombus formation occurring within 5 min at the site of ligation (Suppl. Fig. S2). We found no clear differences in immediate platelet adhesion between the control animals and the dual treated animals (Suppl. Fig. S2). It should be noted that thrombi in the mice receiving dual therapy appeared less dense with a tendency to increased embolization, as observed by visual inspection of intravital microscopy recordings. With respect to vascular damage, temporary ligation of the carotid artery led to localised denudation of the endothelial layer, as can be observed by the absence of nuclei (HE staining, Suppl. Fig. S3A–C, indicated by black arrow) and by the absence of white lining on top of the intima-media layer (SEM, Fig. 1A, indicated by red arrow). One hour post ligation, sub-occlusive thrombi, consisting of > 15 layers of platelets, were observed by electron microscopy in all ligated mouse carotid arteries (Fig. 1A,G) versus no platelet adhesion 3 mm upstream temporary ligation (Fig. 1F). Formed thrombi consisted of a densely packed platelet core and looser outer layer of platelets and retained this size and architecture for at least 24 h (Fig. 1A–D,G). Seventy-two hours post ligation, we observed thrombi (consisting of < 15 layers) in 3 out of 8 mice (Fig. 1E) and a platelet monolayer in the other 5 carotid arteries, restricted to the ligature dimensions (Fig. 1F,G). Independent of the time point, fibrin fibres and red blood cells were sparely observed in the thrombi by means of electron microscopy (Fig. 1A–D). Interestingly, histological analyses do show the presence of red blood cells in around a quarter of the thrombi at 24- and 72-h post ligation, but not at earlier time points (Suppl. Fig. S3C,D).

In the first 24 h post ligation, a loss of cells, presumably smooth muscle cells, was observed in the tunica media (HE staining, Suppl. Fig. S3A–C). In addition, an occasional inflammatory cell (< 10 per tissue section) was found in the media at 3 h post ligation. In the tunica adventitia, an influx of inflammatory cells was found which reached its peak (> 50 inflammatory cells per tissue section) 24 h post ligation and decreased (> 30 inflammatory cells per tissue section) again after 72 h. Necrosis is observed at 24 h post ligation (Suppl. Fig. S3C, indicated by red arrow) but no longer at 72 h. At 72 h post ligation, the intima-media started to remodel at the site of ligation, as can be observed by a disappearance in necrotic tissue and re-appearance of smooth muscle cells (Suppl. Fig. S3D). Furthermore, nuclei started to reappear underneath the thrombi after 72 h in ~ 90% of the stained tissue sections, indicating partial recovery of the endothelial layer (Suppl. Fig. S3D, indicated by blue arrow). Altogether, these data show that temporary ligation of carotid arteries of wildtype mice leads to endothelial denudation and robust and reproducible sub-occlusive thrombus formation. In addition, at the site of ligation an inflammatory response is visible that peaks at 24 h post ligation after which the vessel remodels.

### Effective inhibition of platelet and coagulant activity by aspirin and rivaroxaban treatment

In this experimental study the effect of aspirin and rivaroxaban treatment was investigated on injury-induced vascular remodelling. Treatment was started 4 days prior to ligation, similar for all experimental groups: aspirin, rivaroxaban and aspirin plus rivaroxaban. In all groups, blood pressure and blood cell counts were within the normal range, as measured 14 days after ligation (Suppl. Fig. S4). Furthermore, thrombin-antithrombin (TAT) levels were well below the value of 100 ng/mL in all groups (Suppl. Fig. S5 D), indicating that there was no detectable systemic activation of the coagulation cascade two weeks after temporary ligation nor activation during blood taking^25^. Platelet activation levels were also not elevated at 14 days as platelets from all groups displayed low levels of active integrin α_IIb_β_3_ (JON/A staining: 3 (1–6)% (median [IQR]), data not shown) as determined using whole blood flow cytometry.

The efficacy of in vivo aspirin treatment was checked by measuring the aggregation of washed platelets upon activation by arachidonic acid or collagen. Platelets from aspirin-treated mice were nearly completely inhibited in aggregation with arachidonic acid (Suppl. Fig. S5A), and greatly impaired in collagen-induced aggregation (Suppl. Fig. S5B), indicating maximal inhibition of cylco-oxygenase-1 (COX-1).

Levels of circulating rivaroxaban were determined by measuring the anti-FXa activity in platelet-free plasma samples with a commercial chromogenic assay. Mean levels of plasma rivaroxaban were 368 (258–432) ng/mL (median [IQR]) for the rivaroxaban group, and 417 (231–771) ng/mL for the aspirin/rivaroxaban group, at day 14 after ligation (Suppl. Fig. S5C). There was no correlation between the time of blood drawing, i.e. in the morning versus in the afternoon, and the rivaroxaban levels (data not shown). Taken the differences in binding to plasma proteins and in potency between human and mouse into account^26–28^, these rivaroxaban plasma concentrations translate to clinically meaningful levels of rivaroxaban in the therapeutic range^29,30^. In addition, both rivaroxaban groups showed significantly lower and negligible levels of TAT complexes in comparison to the control and aspirin-treated groups (Suppl. Fig. S5D), also reflecting the impact of FXa inhibition on thrombin formation.

### Treatment with aspirin or rivaroxaban suppressed ligation-induced vascular stiffening and/or intima-media thickening

**Figure 2:**
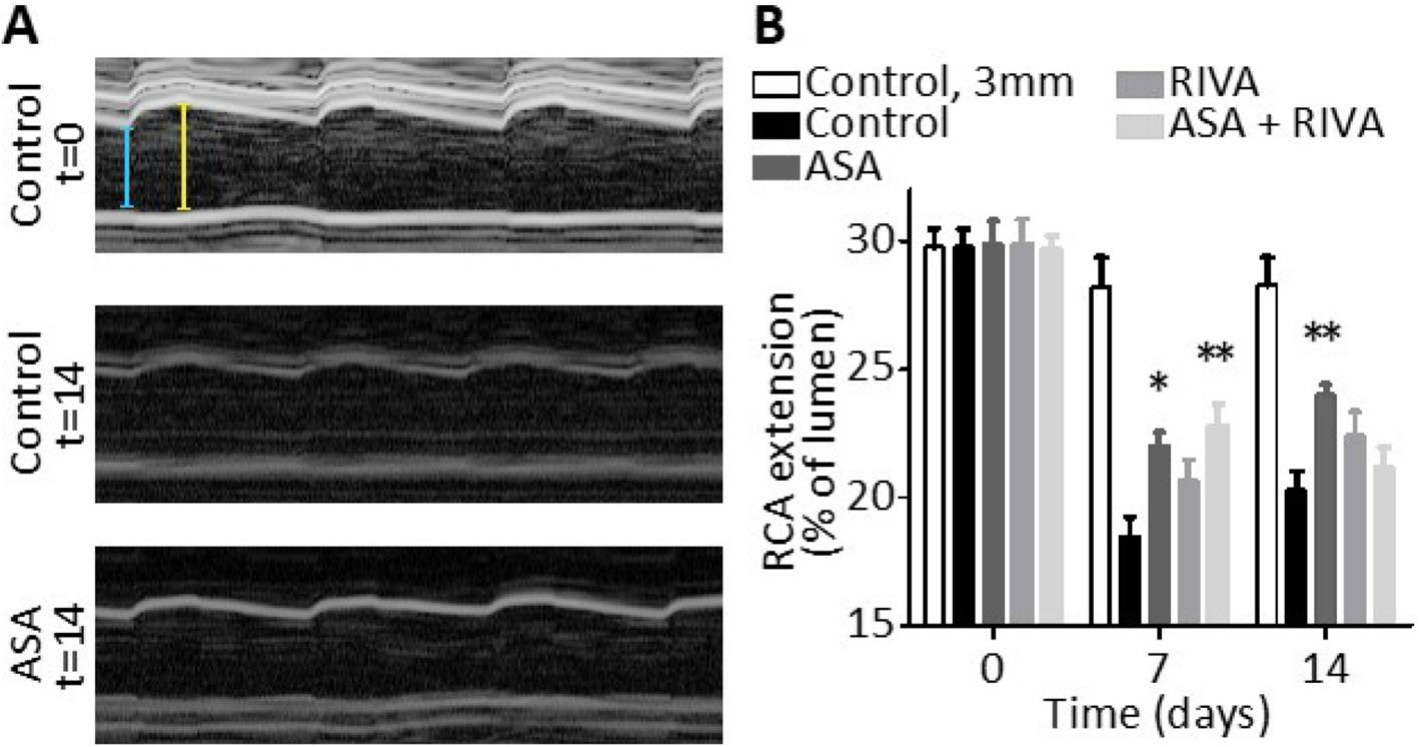
Mouse treatment with aspirin and/or rivaroxaban alleviates ligation-induced vascular stiffening. Carotid vessel wall extension was measured in the four groups of mice using ultrasound (Vevo 2100) at baseline (day 0), 7 and 14 days after temporary ligation. Extension is expressed as % of lumen with quantification done in M-mode. (**A**) Representative images acquired in M-mode, illustrating extension of the carotid artery during three heart beats. Extension was measured from the difference between the yellow and light blue bars. (**B**) Graph of right carotid artery (RCA) extension at site of temporary ligation in control mice or mice treated with aspirin (ASA), rivaroxaban (RIVA) or the combination of both. Also indicated in B are values at three millimetres upstream of injury (control group). Mean ± SEM (n = 11–12), *p < 0.05, **p < 0.01, two-way ANOVA.

Injury-induced vascular remodelling is known to lead to arterial wall stiffening, which is associated with an increased risk for cardiovascular disease^20^. To assess whether aspirin and/or rivaroxaban treatment could alleviate vascular stiffening, vascular extension was measured after temporary ligation using non-invasive echography. The extension was quantified both at the site of ligation and 3 mm upstream, before and after 7 and 14 days of ligation (Fig. 2). After seven days, at the site of ligation a 38% decrease in the extension was found in the control group (Fig. 2B, p < 0.0001), which was nearly absent upstream of the ligation or in sham-treated arteries (Fig. 2B, Suppl. Fig. S6). This suggest local vessel wall stiffening at the site of ligation. Treatment with aspirin (+ 24%, p = 0.0011) and aspirin plus rivaroxaban (+ 19%, p = 0.0108) significantly improved the carotid artery extension at day 7 when compared to control. Treatment with rivaroxaban resulted in a trend towards improved vessel extension when compared to control (T = 7, + 12%, p = 0.25). Interestingly, in the control group the vascular extension reached a nadir after 7 days, after which the extension tended to increase again (Fig. 2B). Such a tendency was also observed in the aspirin and rivaroxaban groups, but unexpectedly not in the group with combined aspirin plus rivaroxaban treatment.

**Figure 3:**
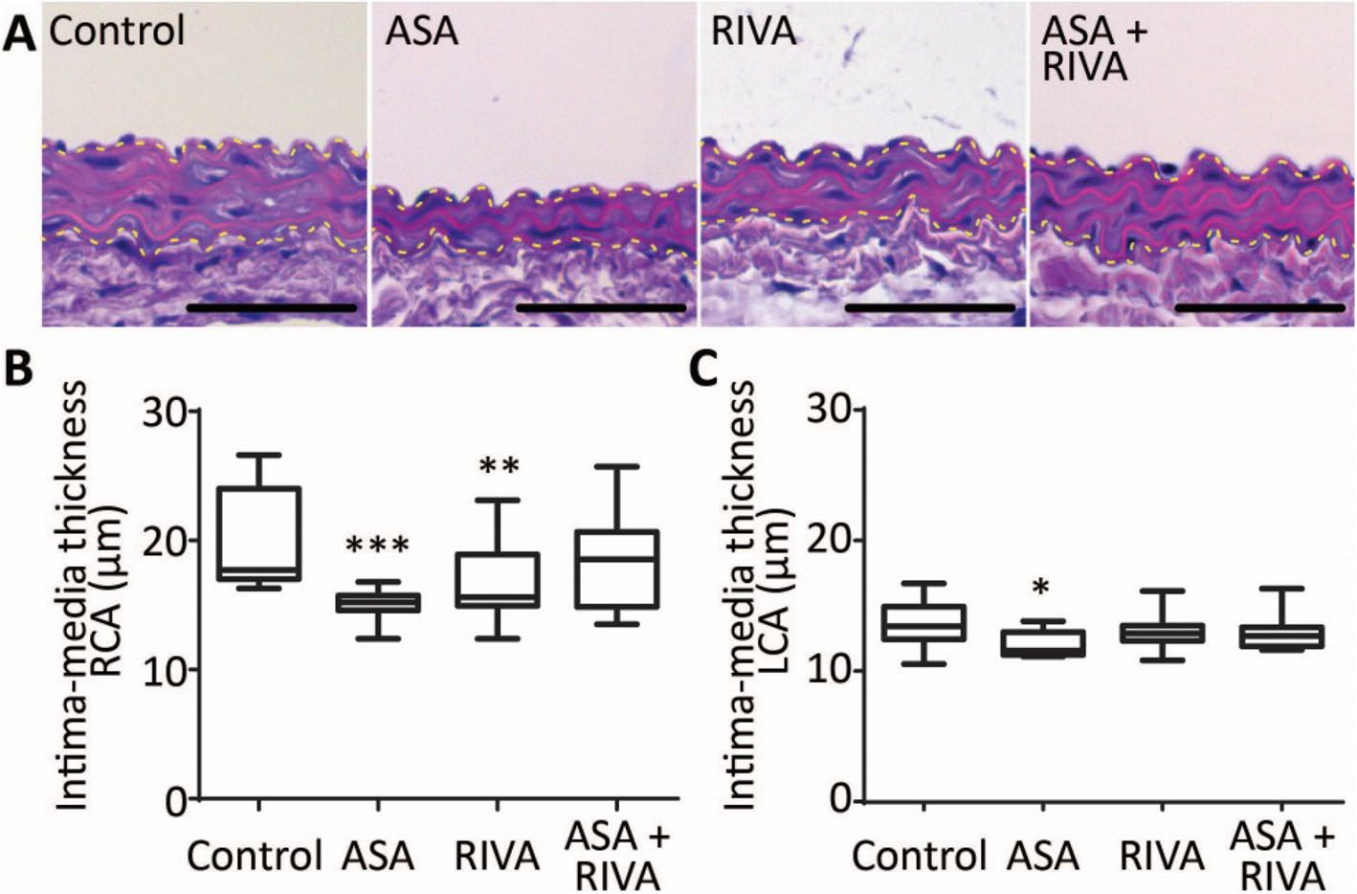
Mouse treatment with aspirin or rivaroxaban reduces post-ligation intima-media thickening. (**A)** Haematoxylin and eosin staining of paraffin sections of the right carotid artery of mice treated with vehicle, aspirin (ASA), rivaroxaban (RIVA) or both, obtained 14 days after temporary ligation. Contours of media are indicated by the yellow dotted line. Bar = 50 µm. Boxplot (min/max) with quantification of media thickness at the site of ligation for the right carotid artery (RCA) (**B**) and left carotid artery (LCA) (**C**). N = 11–12, *p ≤ 0.05, **p ≤ 0.01, ***p ≤ 0.005, one-way ANOVA.

Another important characteristic of vascular remodelling is a changed intima-media thickness of the vessel wall. Histological analysis indicated that, for the control group, the intima-media was 5.9 μm thicker at the site of ligation when compared to the sham-treated left carotid artery (Fig. 3, p = 0.0014). As the intima, after visual inspection, consisted of a single layer of endothelial cells after 14 days, the increased intima-media thickening can be attributed to the media (Fig. 3A). Treatment with aspirin or rivaroxaban alone, but not combined aspirin plus rivaroxaban treatment, significantly decreased this local intima-media thickening (Fig. 3B, 15.1 ± 0.4 μm for aspirin and 16.4 ± 0.9 μm for rivaroxaban vs. 19.6 ± 1.1 μm for control). Observed decreases in intima-media thickness are not caused by changes in cell proliferation as there was no significant change in nuclei count in the media between treatment groups (Suppl. Fig. S7). Interestingly, a small effect of aspirin was observed in the sham-treated arteries (Fig. 3C, 12.1 ± 0.29 μm for aspirin vs. 13.68 ± 0.55 μm for control). Together, these data indicated that at the site of ligation, the vessel wall stiffened, and the intima-media thickened. Aspirin treatment antagonized vascular stiffening and rivaroxaban treatment resulted in a positive trend towards reduced stiffening. Local intima-media thickening was antagonized by both aspirin and rivaroxaban treatment.

### Treatment with aspirin or rivaroxaban affects platelet-leukocyte interaction

**Figure 4:**
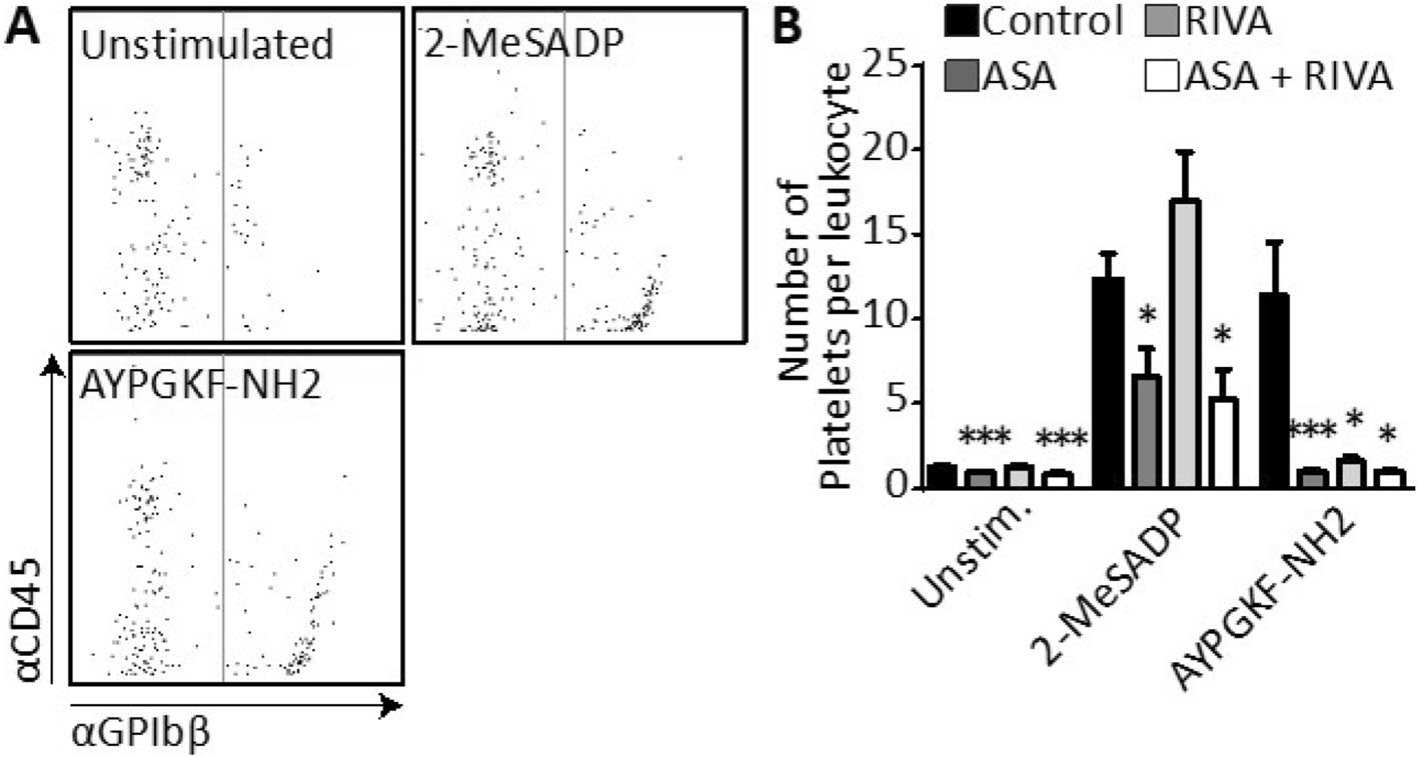
Mouse treatment with aspirin and/or rivaroxaban can inhibit the number of bound platelets per leukocyte. Blood was collected from mice at 2 weeks after temporary ligation of the carotid artery. During the post-ligation period, mice were treated with aspirin (ASA), rivaroxaban (RIVA), ASA + RIVA, or saline (control). Using flow cytometry, platelet-leukocyte aggregates (PLAs) were measured in whole blood by dual staining with APC-αCD45 and DyLight 488-labelled αGPIbβ antibodies. Platelets were unstimulated or stimulated with 2-MeSADP (5 μM) or AYPGKF (30 μM). (**A**) Flow cytometric events were gated for CD45 fluorescence, and size anomalies were excluded by forward sideward scatter analysis. Shown is fluorescence in control blood from all leukocytes without (left of line) and with (right of line) bound platelets as defined by DyLight 488-labelled αGPIbβ fluorescence. (**B**) Calculated number of bound platelets per PLA. Data represented as mean ± SEM, n = 11 (control), 11 (ASA), 12 (RIVA) and 6 (ASA + RIVA) animals per group, *p < 0.05, **p < 0.01, ***p < 0.001, two-way ANOVA.

PLAs within the blood are associated with thrombo-inflammatory diseases, such as cardiovascular diseases^13^. At 14 days after ligation, it was investigated whether the number of PLAs was affected by aspirin and/or rivaroxaban treatment using flow cytometry (Table 1, Fig. 4A, Suppl Fig. S8).

**Table 1:** Inter‑group changes in % of leukocytes with bound platelets (%PLAs) and in number of platelets per leukocyte (# platelets/leukocyte) in unstimulated or 2‑MeSADP‑ or AYPGKF‑stimulated blood samples.

Compared to control group	ASA	RIVA	ASA + RIVA
**%PLAs**			
Unstimulated	↑ p = 0.006	= p = 0.757	↑↑ p = 0.001
2-MeSADP	= p = 0.410	= p = 0.107	= p = 0.300
AYPGKF	= p = 0.277	↓↓ p = 0.002	= p = 0.609
**# Platelets/leukocyte**			
Unstimulated	↓ p = 0.002	= p = 0.454	↓ p = 0.001
2-MeSADP	↓ p = 0.019	= p = 0.206	↓↓ p = 0.015
AYPGKF	↓↓ p = 0.001	↓↓ p = 0.021	↓↓ p = 0.011

= no change, ↓ decreased, ↓↓ > 50% decreased, ↑ increased, ↑↑ > 50% increased.

In unstimulated blood samples from mice, 13.8% of leukocytes were complexed with one or multiple platelets in the control group. In animals receiving aspirin treatment or dual treatment with aspirin plus rivaroxaban levels of PLA were 20.1% or 22.8%, respectively, indicating an almost doubling of PLA levels when compared to the control. Upon rivaroxaban monotreatment PLA levels were unaltered (Table 1). The number of bound platelets per PLA was determined by assuming that the median fluorescence intensity of the glycoprotein marker (αGPIbβ) of single platelet events reflects the fluorescence intensity of one platelet^31^. Absolute values of platelets bound per PLA were low in all unstimulated samples (< 3 platelets per PLA) (Fig. 4B). A relative reduction in the number of platelets per PLA was observed in both the aspirin (− 27%) and the rivaroxaban plus aspirin (− 36%) groups and not in the rivaroxaban group (Table 1, Fig. 4B). In sum, treatment with aspirin, but not rivaroxaban, is associated with an increase in PLA. This increase may be linked to the small but significant rise in platelet count after aspirin treatment, even though the platelets counts in all mice were in normal range (Suppl. Fig. S4A).

To mimic the effect of aspirin and/or rivaroxaban on PLA formation upon platelet activation in vivo, diluted mouse blood samples were incubated with platelet agonists in vitro. Addition of the ADP-P2Y_1/12_ receptor agonist 2-MeSADP to blood from vehicle-treated mice increased the percentage of PLAs by nearly threefold from 13.8 ± 1.4% to 30.2 ± 2.2% (p < 0.0001) and the number of platelets per PLA by 10.1 ± 1.6-fold (Table 1, Fig. 4B). Upon addition of the PAR4 agonist AYPGKF a similar increase in PLAs (from 13.8 ± 1.4% to 29.2 ± 3.7%, p = 0.0021) and number of platelets per PLA was observed (8.7 ± 2.6-fold, Table 1, Fig. 4B). Of note, platelet activation was optimal in the control samples as active integrin α_IIb_β_3_ levels were 60 and 80 ± 2%, respectively, upon addition of 2-MeSADP or AYPGKF. Treatment with aspirin did not affect the increase in percentage of PLAs after agonist stimulation. Yet, rivaroxaban treatment did inhibit the PAR4-mediated increase in percentage of PLAs. Both aspirin and/or rivaroxaban almost completely annulled the PAR4-induced increase in bound platelets per PLA, while only aspirin significantly antagonized the P2Y_1/12_-induced increase for this parameter (Table 1, Fig. 4B). In sum, while rivaroxaban inhibited PAR4-mediated, but not P2Y_1/12_-mediated, increase in PLA levels and number of platelets bound per leukocyte, aspirin was without effect on the number of PLAs formed but did inhibit the number of platelets bound per leukocyte in unstimulated and stimulated blood samples.

### Combined therapy of rivaroxaban and aspirin and rivaroxaban alone reduced thrombus growth and height in vitro

**Figure 5:**
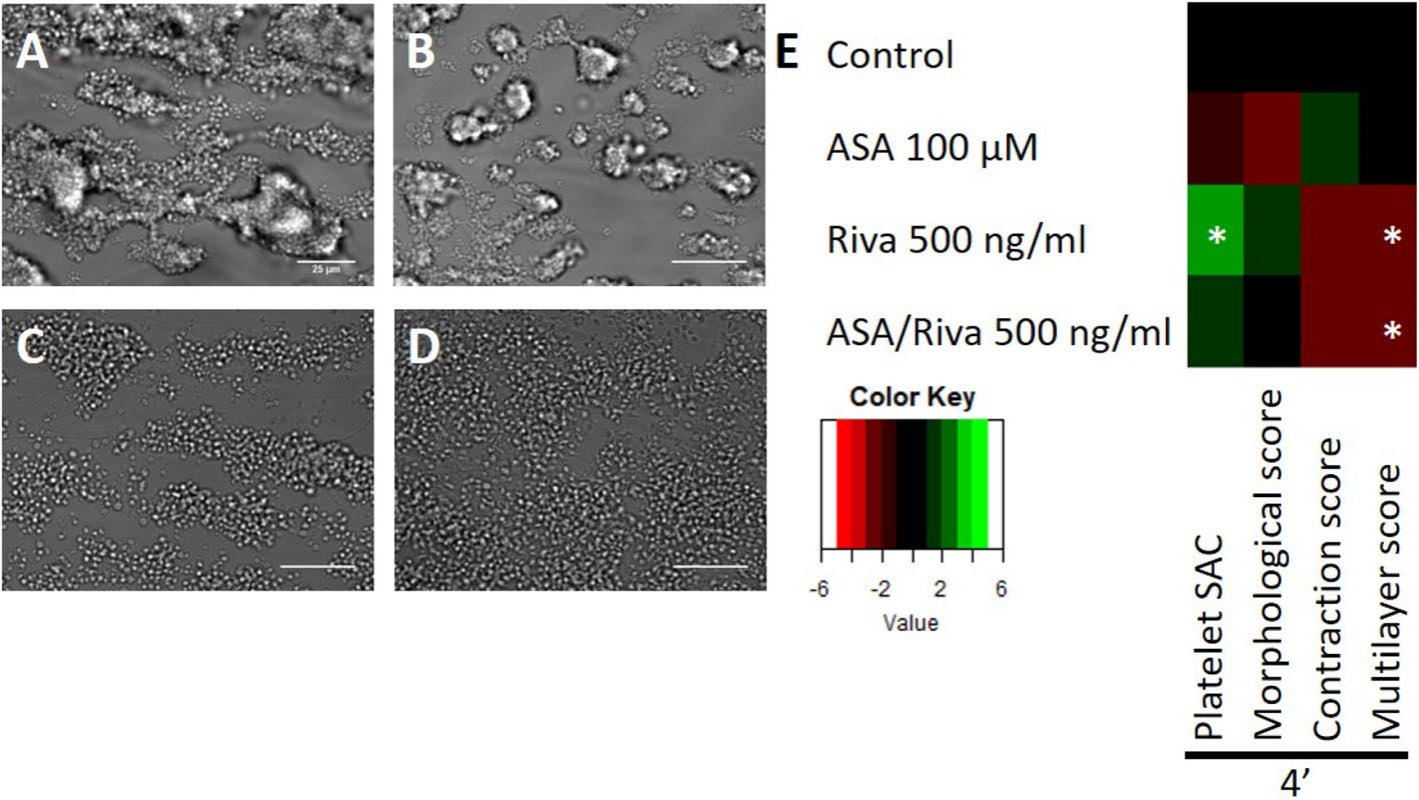
Rivaroxaban treatment results in lower murine thrombi in vitro. (**A**–**D**) Representative brightfield images after 4 min of whole blood perfusion from WT mice. Whole blood was pre-treated with (**A**) saline/DMSO, (**B**) 100 µM aspirin, (**C**) 500 ng/mL rivaroxaban or (**D**) 100 µM aspirin and 500 ng/mL rivaroxaban. Bar = 25 µm. (**E**) Subtraction heatmap of normalised parameters (0–10). Parameters include platelet surface area coverage (%SAC), thrombus morphological score (0–5), multilayer score (0–3) and, contraction score (0–3). N = 9 (control), 4 (ASA), 6 (RIVA) and 6 (ASA + RIVA) animals per group, *p < 0.05 vs. control, Kruskal–Wallis test.

**Figure 6:**
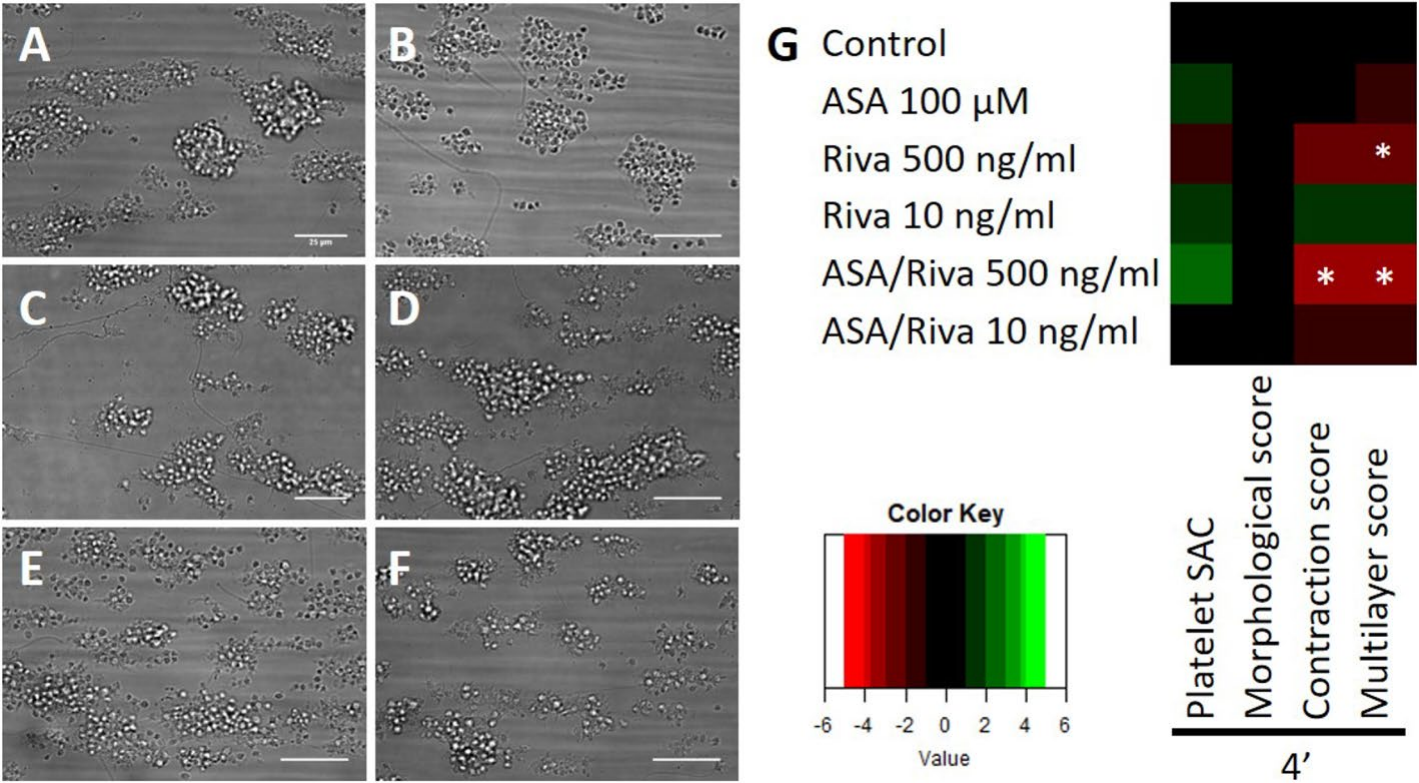
Rivaroxaban treatment results in lower human thrombi in vitro. (**A**–**F**) Representative brightfield images after 4 min of whole blood perfusion from healthy volunteers. Whole blood was pre-treated with (**A**) saline/DMSO, (**B**) 100 µM aspirin, (**C**) 500 ng/mL rivaroxaban, (**D**) 10 ng/mL rivaroxaban, (**E)** 100 µM aspirin and 500 ng/mL rivaroxaban and (**F**) 100 µM aspirin and 10 ng/mL rivaroxaban. Bar = 25 µm. (**G**) Subtraction heatmap of normalised parameters (0–10). Parameters include platelet surface area coverage (%SAC), thrombus morphological score (0–5), multilayer score (0–3) and, contraction score (0–3). N = 14 (control), 5 (ASA), 5 (RIVA 500 ng/mL), 5 (RIVA 10 ng/mL), 5 (ASA + RIVA 500 ng/mL) and 5 (ASA + RIVA 10 ng/mL) per group *p < 0.05 vs. control, Kruskal–Wallis test.

**Figure 7:**
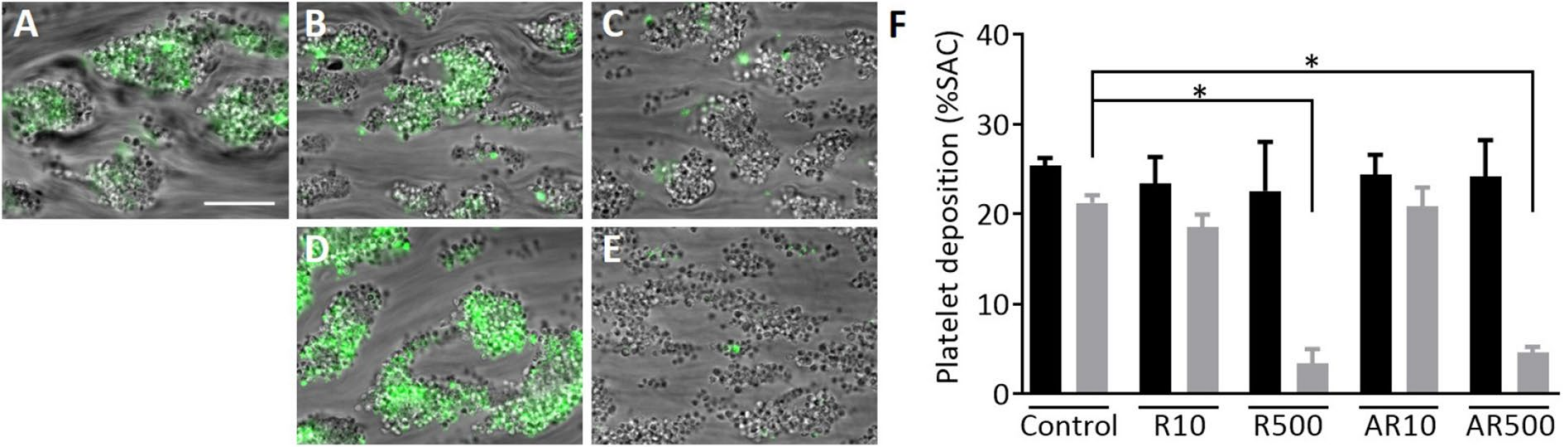
Rivaroxaban inhibits thrombus growth of human thrombi in vitro. Human whole blood was perfused over a combined collagen type I plus tissue factor surface to study thrombus growth under coagulating conditions. Thrombus growth was visualized by DiOC_6_ (0.1 µg/mL) labelled whole blood of the same donor which was pre-treated with 10 ng/mL or 500 ng/mL rivaroxaban with (**B**,**C**) or without (**D**,**E**) 100 µM aspirin or vehicle (**A**). Quantification (%SAC) of platelet deposition for initial thrombus was assessed by brightfield and for thrombus growth by fluorescence microscopy (**F**). Black bar: initial thrombus formation, grey bar: thrombus growth. AR: 100 µM aspirin with 10 or 500 ng/mL rivaroxaban, Ctrl: control, R10: 10 ng/mL rivaroxaban, R500: 500 ng/mL rivaroxaban. Data are represented as bar graphs (median + IQR), n = 4–12, *p ≤ 0.05, **p ≤ 0.01, **p ≤ 0.001, Kruskal–Wallis test.

To assess the effect of aspirin and/or rivaroxaban on thrombus formation a series of in vivo intravital microscopy studies and in vitro microfluidic flow assays were conducted. For the in vivo experiments, temporary ligation was inflicted as described for the intervention study. Unfortunately, the relatively small size of the thrombi (25–30 µm in height, based on Fig. 1A) combined with the limited resolution of the intravital microscope used limited detecting any potential differences in thrombus size upon aspirin and rivaroxaban treatment in comparison to control (Suppl. Fig. S2). To address this limitation and to examine the thrombus formation process in extensive detail, in vitro experiments were carried out with whole blood of C57BL/6 mice to which 100 µM aspirin and/or 500 ng/mL rivaroxaban was added for 10 min. Blood was recalcified and perfused over a collagen type I surface at a calculated arterial wall shear rate of 1000 s^−1^. When comparing aspirin treatment to vehicle controls, a trend was observed towards unstable, smaller (morphological score, 2D) and lower (multilayer score, 3D) thrombi (Fig. 5A,B,E). Inhibition of FXa with rivaroxaban resulted in a more profound inhibition of the thrombus forming process with a lower thrombus height (p = 0.0190), a larger surface area (p = 0.0365) covered with platelets when compared to controls, and a tendency to reduced platelet contraction (p = 0.052) (Fig. 5A,C,E). Dual treatment gave a similar but overall, less profound effect than rivaroxaban on thrombus formation (Fig. 5D,E). Besides using mouse blood, the effects of aspirin and rivaroxaban were also assessed on thrombus formation using human blood, obtained from healthy donors via venepuncture (Fig. 6). Similar as observed in mouse, treatment with rivaroxaban (500 ng/mL) resulted in a decreased thrombus contraction and multilayer score with human blood (Fig. 6D,F,G). Thrombus growth was examined in a separate experiment and found to be inhibited by rivaroxaban but not by aspirin (Fig. 7). A 50-times lower dosage of rivaroxaban did not significantly affect the thrombus forming process, nor did 100 µM aspirin (Figs. 6, 7). In sum, in this microfluidic setup, rivaroxaban reduced thrombus growth and height whereas aspirin showed a trend towards reduction.

## Discussion

Here we assessed the effects of aspirin and rivaroxaban on thrombus formation and vascular remodelling upon temporary ligation of the mouse carotid artery. We found that ligation of the carotid artery consistently caused localised endothelial cell denudation and the formation of sub-occlusive platelet-rich thrombi. At the site of ligation, the carotid artery remodelled as evident from a reduced vessel wall extension—thus implying a local vessel stiffening—and a thickened intima-media layer. Aspirin treatment antagonized vascular stiffening and rivaroxaban treatment led to a trend towards reduced stiffening. For humans, conflicting reports exist on the effects of aspirin or rivaroxaban intake on arterial stiffness^20–24,32–34^, whereas in mice this appears not to have been studied at all. In humans, aspirin treatment can prevent arterial stiffening caused by an acute systemic inflammation^23^ or by smoking^32,34^. Interestingly, variable results are obtained for small scale clinical studies with respect to an effect of aspirin on vascular stiffening: a reducing effect of aspirin treatment was reported in a group of 15 patients with hypertension versus 15 controls^22^, but no effect in 21 diabetic patients versus age and sex matched controls^33^. Furthermore, no difference in arterial stiffness was found between subjects with CAD that are aspirin sensitive versus aspirin resistant^21^. There is one study reporting that switching from warfarin to rivaroxaban, in patients with atrial fibrillation, was associated with a decreased pulse wave velocity and vascular stiffening^24^. Knowing that arterial wall stiffening is associated with an increased risk for cardiovascular disease^20^ and the potential vascular protective of aspirin and rivaroxaban, it would be of great value to investigate the effect of aspirin and/or rivaroxaban on vascular stiffening in large scale clinical studies.

Intima-media thickness of the carotid artery reflects the presence and progression of atherosclerosis in humans^35^. In the present study, it is demonstrated for the first time that both aspirin and rivaroxaban suppress vascular injury-induced intima-media thickening of the mouse carotid artery. In addition, in absolute numbers a small (on average -1.5 µm) but nonetheless significant effect of aspirin on intima-media thickness of the sham-treated carotid artery was detected. Knowing that aspirin blocks COX-1-mediated thromboxane A_2_ (TxA_2_) synthesis in platelets and that TxA_2_ is not involved in platelet adhesion to a damaged or inflamed vessel wall^36^, it is likely that the smaller intima-media thickness of the undamaged artery upon aspirin treatment is caused by a direct effect of aspirin on the vessel wall. Next to irreversibly blocking COX-1, aspirin modifies the activity of COX-2. In contrast to COX-1, which produces prostaglandins that are involved in physiological process, COX-2 activity leads to the generation of prostaglandins that mostly act proinflammatory, like PGE2^37^. A daily intake of 100 mg of aspirin is regarded as sufficient to completely block TxA_2_ formation and with increasing dosages COX-2 activity is further inhibited without providing an additional anti-platelet effect^38^. In this study, the daily intake of aspirin was estimated to be 3 mg/kg/day, which would correspond to 210 mg daily for an average person of 70 kg. In sum, the effect of aspirin on the vascular remodelling in the present study is caused by a combination of full inhibition of TxA_2_ generation and partial inhibition of COX-2-mediated prostanoid synthesis.

Determining PLAs is considered to be of potential diagnostic and/or prognostic value for a range of thrombo-inflammatory diseases, like cardiovascular diseases^39^. Platelet-leukocyte crosstalk fosters leukocyte recruitment and extravasation to inflammatory sites and stimulates leukocytes release of pro-inflammatory mediators, as reviewed by Finsterbusch et al.^39^. Rivaroxaban treatment was not associated with a difference in PLA or number of platelets per leukocyte in unstimulated and P2Y_1/12_ stimulated blood, but did suppress the PLA formation upon PAR-4 stimulation in vitro, which is in agreement with a report where rivaroxaban had an attenuating effect on platelet-leukocyte-endothelial interaction in the mouse microcirculation upon vascular injury^40^. Given these findings, examining the effect of rivaroxaban on PLA in patient samples would be of great interest. In healthy subjects and patients with cardiovascular disease, aspirin was found to be less potent than P2Y_12_ inhibition, or possibly without effect, in reducing platelet-leukocyte aggregate formation^16,17^. Unexpectedly, in the present study, treatment with aspirin was linked to an increase in the number of PLA when compared to the control group. A potential explanation might be the rise in platelet count, albeit still in the normal range, after aspirin treatment. Interestingly, in both aspirin groups a lower number of platelets per leukocyte were found in comparison to the control group. Currently, there are (almost) no reports on the number of platelets per leukocyte in patients as the absolute number of platelets cannot be directly assessed using conventional flow cytometry. For our study, these numbers were estimated based on the fluorescence intensity of one platelet. With ‘imaging flow cytometry’ such direct measurements are possible. Furthermore, if and how the number of platelets affects leukocyte function would be interesting to study.

The rivaroxaban levels measured in the current study were within the clinical therapeutic range when translated to the human situation^29,30^. Combined with the negligible TAT levels upon treatment with rivaroxaban, this suggests that FXa activity was adequately inhibited. For rivaroxaban to exert its effect a certain extent of coagulant activity should be present in the model system used. Earlier studies demonstrated platelet-collagen interactions to be the major determinant of arterial thrombus formation upon a temporary ligation of 5 min of the carotid artery of healthy mice^41^, and also observed tissue factor- and factor XII-dependent fibrin formation in this model^42,43^. Our model of temporary ligation of the carotid artery consistently caused localised endothelial cell denudation and formation of sub-occlusive platelet-rich thrombi. The extent of coagulant activity in our in vivo model appears to be milder, with no large fibrin fibres being detectable in formed thrombi, when compared to the work of Massberg et al. and Reinhardt et al.^41,42^. The difference in extent of coagulant activity might be linked to a shorter duration of the ligation of 2 times 60 s in our study versus 5 min in the earlier work of Massberg and Reinhardt et al.^41,42^. We do find that the thrombi display a dense thrombus core, suggestive of FXa activation, thrombin generation and thus some coagulant activity^44^. This suggests that the mitigating effect of rivaroxaban on intima media thickening and trend towards reduced vascular stiffening in this study is likely more pronounced in experimental models with more coagulant activity.

Using our multiparameter microfluidics assay^45^, we are the first to demonstrate that rivaroxaban treatment results in reduced in vitro thrombus growth and height using both mouse and human blood. These data extent work by others who demonstrated that rivaroxaban reduces in vitro thrombus formation in human blood^46–48^. Our data show a trend towards a reduced thrombus contraction in vitro with rivaroxaban. This may have potential clinical significance as a high-tensile platelet fibrin clot strength is associated with the occurrence of ischemic events after in-stent restenosis and elective coronary stenting^49,50^. Given the proven clinical benefit of aspirin in secondary prevention of cardiovascular, the present lack of effect of aspirin on whole blood thrombus formation in vitro comes across as unexpected. It is however consistent with the variable effect of aspirin that is observed in microfluidics, which range from reduced thrombus formation to no effect, as reviewed by van Kruchten et al.^51^ and by Brouns et al.^52^. Although detailed insight into the underlying reasons for these variable results of aspirin on in vitro thrombus formation is still lacking, differences in microfluidic setup, such are collagen concentration^53^, temperature and shear rate/perfusion time^53–55^, are likely important underlying causes.

The experimental setup used for this study has several limitations. Temporary ligation of the carotid artery can damage all vessel wall layers and most likely is the cause of the loss of smooth cells in the media layer and the influx of inflammatory cells in the adventitia 24 h post-ligation. Although regrowth of cells in the media was observed 3 days post-ligation, it is likely that the vascular stiffening and intima-media thickening, measured at two weeks post-ligation, partly stems from the vessel wall damaging effect of the ligature. The latter does not exclude a role for platelets and the coagulation system in vascular remodelling. Clearly, the endothelium was denudated upon ligation, which led to sub-occlusive thrombus formation. A vascular protective effect of aspirin and of rivaroxaban was observed. In absolute numbers, the observed effects were small but detectable. We speculate that in mice with an atherosclerotic background, aspirin and rivaroxaban will have an even more profound vascular protective effect. The potential vascular protective effect of FXa inhibition is related to multiple mechanisms. Next to its main role in thrombin generation, FXa exerts direct pro-inflammatory effects on the vasculature by binding to PAR receptors^4^. As described above, the aspirin dosage that was used here fully inhibits platelet TxA_2_ formation but also has direct anti-inflammatory effects on the vessel wall mediated via COX-2 inhibition. It should be noted that maximally blocking TxA_2_ generation without interfering with the production of other prostanoids is not possible when using aspirin^38^. In order to dissect the platelet-mediated effects of aspirin from the vessel wall mediated ones, a tissue (or platelet) specific COX knock-out mouse model could be used. Recently, such mice have become available^56^.

## Materials and methods

### Characterization of vascular damage and thrombus formation upon temporary ligation

An experimental model was set up to study injury-induced vascular remodelling in mice upon temporary ligation of the carotid artery. To characterize the extent of the vascular damage, and the extent and reproducibility of thrombus formation upon ligation, intravital and scanning electron microscopy was performed, as described below. All animal experiments were approved by the Animal Research Ethics Committee of Maastricht University. All experiments were performed in accordance with relevant guidelines and regulations. Male C57BL/6 mice (16 weeks old) were obtained from Charles River (Sulzfeld, Germany), and were fed chow diet. Four days before temporary carotid artery ligation all mice were switched to a western type diet supplemented with 0.25% cholesterol.

#### Temporary ligation of the carotid artery

Mice were anaesthetized by subcutaneous injection of 75 mg/kg ketamin and 1 mg/kg medetomidin. Body temperature was held at 37 °C. The left and right carotid arteries were carefully dissected free from surrounding tissue. A ligature was then placed around the right carotid artery one millimetre upstream of the bifurcation using a Pronova (polyhexafluoropropylene-VDF) suture (70 µm Ethicon, Somerville, NJ, USA), which was tightened for 60 s, released and then immediately again tightened for 60 s. A suture was also applied around the left carotid but was not tightened (sham). After removal of the ligature sutures, the surrounding tissue was repositioned, and the skin layer was closed with 5-0 sutures. The animals were then subcutaneously injected with antidote 1 mg/kg atipamezole (Orion Pharma, Espoo, Finland) and 0.1 mg/kg buprenorphine (Animalcare, York, United Kingdom), and were allowed to recover at 28 °C. Injection of the opioid buprenorphine was repeated 24 h after surgery.

#### Intravital microscopy

Ligation-induced thrombus formation was visualized with an intravital microscope (Leitz, Jena, Germany), essentially as described^57^. Thirty minutes before ligation, the animals were injected intravenously with rivaroxaban (0.3 mg/kg) and aspirin (5 mg/kg) or vehicle (saline). Five minutes prior to ligation, the animals were injected with 5,6-carboxyfluorescein diacetate succinimidyl ester (CFSE, Thermo Fisher Scientific, Waltham, MA, USA)-labelled platelets, obtained from a donor mouse, as described before^58^. Upon ligation, thrombus formation was directly (< 60 s) visualized, by capturing 12-bit fluorescence images at 33 Hz, using a back-thinned electron multiplier C9100-12 EM-CCD camera (Hamamatsu, Hamamatsu City, Japan) at fixed gain settings.

#### Scanning electron microscopy and histology

Mice were euthanized by anaesthetics overdose at T = 1, 3, 6, 24 or 72 h after temporary ligation. Carotid arteries were immediately dissected free from surrounding tissue and fresh 4% paraformaldehyde in PBS was applied on top of the carotids for 15 min. The carotids were deliberately not flushed with fixative in order to prevent washing away (parts of) the thrombi. Carotids were gently removed and incubated overnight in 4% paraformaldehyde and processed as described for histology. Coupes were either deparaffinised in xylene and dried overnight, sputter coated in gold (Q150RS, Quorum Technologies Ltd, Laughton, UK) and visualized with a scanning electron microscope (Phenom XL, Phenom-World, Eindhoven, The Netherlands) at 10 kV, or deparaffinised in xylene and stained for haematoxylin and eosin (HE, Sigma-Aldrich, St. Louis, MO, USA) to examine vascular damage and inflammatory cell influx.

### Intervention study

All animal experiments were approved by the Animal Research Ethics Committee of Maastricht University. All experiments were performed in accordance with relevant guidelines and regulations. The intervention study, i.e. studying thrombus activities and vascular remodelling upon temporary ligation of the mouse carotid artery, is schematically depicted in Suppl. Fig. S1. Below, the materials and methods of the study are described in chronological order.

#### Mouse treatment with aspirin and/or rivaroxaban

Male C57BL/6 mice (16 weeks old) were obtained from Charles River (Sulzfeld, Germany), and were fed chow diet. Four days (T =  − 4) before temporary carotid artery ligation all mice were switched to a western type diet supplemented with 0.25% cholesterol and treated with aspirin, rivaroxaban, aspirin plus rivaroxaban, or vehicle. Acetylsalicylic acid (aspirin, ASA) was provided in drinking water (75 mg/L, Aspegic, Sanofi, France) ad libitum. The aspirin-containing drinking water was refreshed daily and total aspirin intake in individual mice was calculated at approximately 3 mg/kg/day. In a separate set of experiments, the efficacy of aspirin to inhibit aggregation of murine platelets was measured by light transmission aggregometry. Washed platelets (2 × 10^8^/L), from aspirin-treated mice, were obtained as described before^59^. Aggregation of recalcified (2 mM CaCl_2_) washed platelets was induced by collagen (0.8 µg/mL, Horm, Nycomed Austria, Linz, Austria) or arachidonic acid (12.5 µM, BioData, Boston, MA, USA) under constant stirring at 37 °C, using a lumi-aggregometer (Chronolog Corporation, Havertown, PA, USA).

Rivaroxaban (RIVA, Bayer AG, Wuppertal, Germany) was mixed with the diet at 1.2 mg/g chow. This concentration is optimized to induce a similar reduction in thrombin generation in mice plasma as in human plasma (unpublished data, courtesy of Dr. Henri Spronk). The rivaroxaban concentration was determined in mouse platelet-free plasma (obtained by centrifugation at 22,500×*g* for 10 min), with a chromogenic anti-factor Xa assay according to the manufacturer’s instructions (Biophen Direct Factor Xa Inhibitor, HYPHEN BioMed, Neuville Sur Oise, France). The efficacy of rivaroxaban treatment was assessed by a thrombin-antithrombin (TAT) complex assay, according to the manufacturer’s instructions (Enzygnost TAT micro, Siemens Healthcare, Marburg, Germany). Vehicle-treated animals (control group) received the western diet and normal drinking water ad libitum. Pharmacological treatment was continued post-ligation for 2 weeks after which the mice were sacrificed, and blood and carotid vessels were collected for analysis.

#### Non-invasive measurement of carotid artery extension and blood pressure

Murine carotid artery extension was examined non-invasively by ultrasound before ligation, and at one and two weeks after ligation using the Vevo 2100 small animal imaging platform (Visual Sonics, Toronto, ON, Canada). For this measurement, anaesthesia was induced in the mice by 3–4% isoflurane and maintained by 1.5–2.5% isoflurane, in order to support regular heartbeat and breathing. Hair around the neck was shaved and chemically depilated. The animals were placed on a heated platform (37 °C) in supine position. Rectal temperature and heart rate were monitored throughout the procedure. A high frequency MS-700 ultrasound scan head (50 MHz) was used, which allowed a lateral resolution of 0.5 µm and frame rates up to 1400 frames per second. B-mode was employed to visualise the common carotid artery at one and three millimetres proximal of the carotid bifurcation. Carotid extension was measured during five separate heartbeats using the M-mode and was defined as the difference in carotid lumen diameter between systole and diastole. Blind analysis was performed by two independent researchers. Diastolic and systolic blood pressures were measured 2 weeks after the ligation intervention, by placing a tail-cuff around the mouse tail and recording volume pressure (Coda 6, Kent Scientific Corporation, Torrington, CT, USA).

#### Blood cell counts and determination of platelet-leukocyte complexes

Two-weeks post-ligation, blood was obtained via the vena cava of anaesthetized mice, and anticoagulated with citrate (12.9 mM trisodium citrate, final concentration). Blood cell counts were measured using an automated cell count analyser (Sysmex XP-300, Lincolnshire, IL USA). Normal ranges of blood cells were taken from reference values provided by Charles River and Jackson laboratories (Wilmington, MA, USA and Bar Harbor, ME, USA). PLAs were detected using flow cytometry within 1 h after blood taking. Therefore, whole blood was diluted 25 times in Tyrode’s HEPES (TH) buffer (136 mM NaCl, 2.7 mM KCl, 0.42 mM NaH_2_PO_4_, 5 mM HEPES, 2 mM MgCl_2_, 0.1% (w/v) glucose and 0.1% (w/v) bovine serum albumin (BSA), pH 7.45). The diluted samples were recalcified with 2 mM CaCl_2_ in the presence of 20 U/mL fragmin and 20 µM PPACK, and then stimulated with 5 µM 2-methylthio-ADP (2-MeSADP) (Santa Cruz Biotechnology, Dallas, TX, USA), 30 µM PAR4-activating peptide (AYPGKF, Bachem Biosciences, Bubendorf, Switzerland) or vehicle, under stirring conditions for ten minutes at 37 °C. Aggregates of leukocytes and platelets were detected with APC-conjugated anti-CD45 antibody (10 µg/mL, eBioscience, San Diego, CA, USA) and DyLight488-anti-GPIbβ antibody (20 µg/mL, Emfret Analytics). After 15 min incubation at room temperature, blood samples were 300 times diluted in TH buffer, and subsequently analysed with a BD Accuri C6 flow cytometer (Becton Dickinson, Franklin Lakes, NJ, USA). PLAs were defined as CD45-positive events that were also positive for DyLight488 and had the pre-defined forward/side scatter characteristics. The median intensity of the DyLight488-positive platelet cloud was considered to represent the fluorescence of one platelet. The DyLight488 intensity of PLA was divided by this median to get an estimate of the number of platelets bound per aggregate^31^. Platelet activation was assessed with antibodies against activated integrin α_IIb_β_3_ (12 µg/mL, PE-labelled JON/A antibody) and P-selectin (12 µg/mL, FITC-labelled anti-CD62P antibody, both Emfret Analytics, Würzburg, Germany). Blind analysis was performed by two independent researchers.

#### Immunohistochemistry

Two weeks after the temporary ligation intervention, the mice were anaesthetized by subcutaneous injection of ketamin and medetomedin and blood was drawn from the vena cava as described above. The vena cava was then severed, while slowly injecting 5 mL 1% nitroprusside (Janssen Chimica, Geel, Belgium) into the right ventricle, followed by 5 mL 4% paraformaldehyde (Merck, Kenilworth, NJ, USA). The carotid arteries were excised and fixed in 4% paraformaldehyde during 24 h, and then paraffin embedded. Serial cross section (4 µm) were cut and the carotid arteries were examined after histological staining at the site of ligation. Media thickness was determined after staining the coupes with HE. Media thickness was determined as an average of eight evenly distributed measuring points around the circumference of one carotid artery at the site of ligation. Image analysis was performed using a LAS AF Lite v2.6.3 microscope (Leica, Wetzlar, Germany). Blind analysis was performed by two independent researchers.

### Characterization of thrombus formation in vitro

Whole blood thrombus formation studies were performed in vitro, using microfluidics^45^, to characterize the effect of aspirin with(out) rivaroxaban on thrombus activities in more detail. Below the materials and methods of these in vitro studies are described.

#### Blood drawing

Blood was obtained from male mice with C57BL/6 background that were fed chow diet. Mice were anaesthetized by subcutaneous injection of either ketamine (100 mg/kg), xylazine (12.5 mg/kg) and atropine (125 µg/kg) or ketamine (75 mg/kg) and medetomidine (1 mg/ kg) or inhalation of isoflurane gas after which the blood was collected via the inferior vena cava using 11 µM trisodium citrate as anticoagulant. The change in anaesthetics was caused by a change in policy in the local animal house. Blood samples were kept at room temperature and used within 2 h. All animal experiments were approved by the Animal Research Ethics Committee of Maastricht University. All experiments were performed in accordance with relevant guidelines and regulations.

Alternatively, blood was obtained from healthy volunteers free from antiplatelet and anticoagulant medication for at least two weeks. All healthy volunteers gave full informed consent for participation according to the Helsinki declaration. Permission was obtained from local Medical Ethical Committee (METC 10-3-023, Maastricht University). All experiments were performed in accordance with relevant guidelines and regulations. Blood was drawn via venepuncture using a vacuum container (BD, Franklin Lakes, NJ, USA) and collected into 9 mL tubes containing 3.2% trisodium citrate. Blood samples were kept at room temperature and used within 5 h.

#### Preparation of microspot coating

Glass coverslips (24 × 60 mm #1, Thermo-Fisher, Waltham, MA, USA) were cleaned and degreased then coated with 5 µL mixture of Horm collagen type I (50 µg/mL, Takeda Austria GmbH, Linz, Austria) and dye-quenched (DQ) collagen (50 µg/mL, type I collagen from bovine skin, fluorescein-conjugated, Invitrogen, Carlsbad, California, USA) or collagen type I (50 µg/mL) co-coated with tissue factor (500 pM, Innovin, Dade Behring, Deerfield IL, USA). Coated glass coverslips were blocked with either Tyrode HEPES (TH) buffer pH 7.45 (136 mM NaCl, 5 mM HEPES, 2.7 mM KCl, 0.42 mM NaH_2_PO_4_·6H_2_0) containing 1% BSA for mouse whole blood perfusion assay or HEPES buffer pH 7.45 (136 mM NaCl, 10 mM HEPES, 2.7 mM KCl, 2 mM MgCl_2_) containing 1% BSA for human whole blood perfusion assay. Coverslips were then mounted onto a transparent flow chamber (height 50 µm, width 3.0 mm, length 30 mm), air-tight clamped into a holder and pre-rinsed with TH buffer pH 7.45 (2 mM MgCl2, 0.1% glucose and 0.1% BSA) or HEPES buffer (0.1% glucose and 0.1% BSA), respectively.

#### Whole blood perfusion assay

Mouse and human blood samples were pre-incubated for 10 min with aspirin (100 µM), rivaroxaban (10 and 500 ng/mL) or both. Aspirin was dissolved in saline and rivaroxaban in DMSO, therefore vehicle controls were pre-incubated with 0.018% saline, 0.0005% DMSO or both, respectively. Mouse and human blood were recalcified (7.5 mM CaCl_2_, 3.5 mM MgCl_2_ in TH buffer pH 7.45 or 6.3 mM CaCl_2_, 3.2 mM MgCl_2_ in HEPES buffer pH 7.45) prior to entering the microfluidic chamber using two pulse-free micro-pumps and y-shaped mixing tubing^60^. Mixing was at a volume ratio of 9 (blood) to 1 (recalcification medium). Mouse citrated whole blood was perfused for four minutes and human citrated whole blood for six minutes over a collagen type I surface, both at a calculated arterial wall shear rate of 1000 s^−1^. Platelet adhesion and thrombus formation were monitored during whole blood perfusion. Whole blood perfusion was followed by two minutes of rinse buffer (5 mM CaCl_2_, 5 mM MgCl_2_, 0.25 mg/mL GPRP and TH or HEPES 7.45 buffer) containing aspirin, rivaroxaban, saline or DMSO, respectively. Image recording was performed with confocal Zeiss microscope (Plan-Apochromat 63x/1.40 Oil DIC M27 objective, Carl Zeiss AG, Oberkochen, Germany) and EVOS fluorescence microscope (Olympus UPLSAPO 60x oil-immersion objective, Life Technologies, Carlsbad, CA, USA). Images were collected at 8-bit (1388 × 1040 or 1360 × 1024 pixels).

In a separate set of experiments, thrombus growth was specifically studied. For this purpose, recalcified human whole blood was first perfused for 3 min over a collagen type I surface co-coated with tissue factor for initial thrombus formation. After a rinse with autologous plasma for 2 min, DiOC_6_ (0.1 µg/mL, AnaSpec, Fremont, CA, USA) labelled whole blood of the same donor was perfused for 2 min to visualise thrombus growth. All perfusions were at a calculated arterial wall shear rate of 1000 s^−1^. GPRP (final concentration 0.25 µg/mL) was added to whole blood and plasma. When indicated, plasma and secondary blood perfusion were pretreated with 10 ng/mL or 500 ng/mL rivaroxaban with or without 100 µM aspirin for 10 min. Image recording was performed with EVOS fluorescence microscope. Representative brightfield and fluorescence microscopic images were captured using Olympus 60 × oil-immersion objective and GFP 470 nm LED diode cube. Images were collected at 8 bits (1360 × 1024 pixels). Blind analysis was performed.

### Statistics

Statistical analyses were performed with GraphPad Prism 8 (GraphPad Software, San Diego, CA, USA). Data were checked for normality with the D’Agostino Pearson omnibus normality test. Significance of differences were determined by Kruskal–Wallis test or ANOVA analysis of variance followed by a (uncorrected) Dunnett’s or Tukey post-hoc test, as appropriate. Numerical data of interventions are presented as means with SD. p values < 0.05 were considered to be significant. For comparative data analysis in heatmaps, mean values were linearly normalized to a range from 0 to 10 (Microsoft Excel). Colour key of subtraction heatmap of scaled values only shows values between − 6 and 6. Values < average − 2SD or values > average + 2SD were considered significant.

## Supplementary information

Supplementary Information.
