# Supplementary material for: Vascular protective effect of aspirin and rivaroxaban upon endothelial denudation of the mouse carotid artery
Source: Sci Rep. 2020 Nov 9;10:19360. doi: 10.1038/s41598-020-76377-8 (PMC7653917; doi:10.1038/s41598-020-76377-8)
Supplement: Supplementary file 1 — Supplementary Information. [file 41598_2020_76377_MOESM1_ESM.docx]

**Vascular protective effect of aspirin and rivaroxaban upon endothelial denudation of the mouse carotid artery**

**Running head:** Role of aspirin and rivaroxaban in thrombus formation and arterial vessel wall remodelling.

Mastenbroek T.G.^1,4*^, Karel M.F.A.^1*^, Nagy M.^1^, Chayoua W.^1,2^, Korsten E.I.J. ^1^, Coenen D.M. ^1^, Debets J.^3^, Konings J. ^2^, Brouns A.E.^3^, Leenders P.J.A.^3^, van Essen H.^3^, van Oerle R.^1^, Heitmeier S. ^5^, Spronk H.M.^1^, Kuijpers M.J.E.^1^, Cosemans J.M.E.M.^1^

Department of Biochemistry^1^, Synapse Research Institute^2^ and Department of Pharmacology & Toxicology^3^, Cardiovascular Research Institute Maastricht (CARIM), Maastricht University, Maastricht, the Netherlands. Department of Complex Tissue Regeneration, MERLN Institute for Technology-Inspired Regenerative Medicine, Maastricht, the Netherlands^4^. Cardiovascular Research Institute, Bayer AG, Wuppertal, Germany^5^.

*Equal contribution

**Supplemental Figures**

**
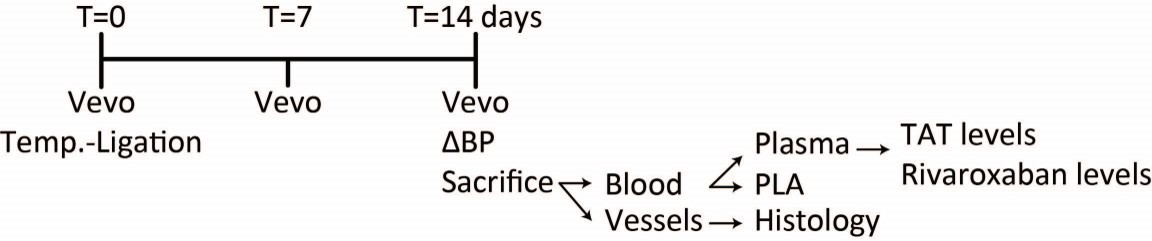
**

**Supplemental figure 1. Timeline of the *in vivo* study.** Ultrasound examination of carotid extension was measured with a Vevo 2100 system. Ligation of the carotid and blood and tissues were processed as indicated in the Materials and methods section. BP, blood pressure; PLA, platelet leukocyte aggregate; TAT, thrombin‑antithrombin.


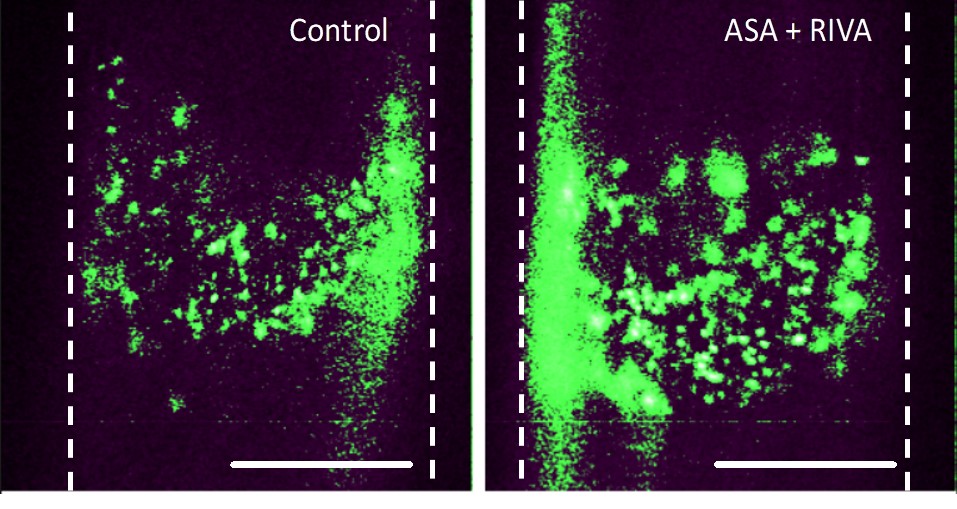


**Supplemental figure 2. Temporary ligation of the carotid artery provokes consistent thrombus formation *in vivo*.** Mice were injected with CFSE labelled donor platelets prior to temporary ligation (note that only 10-20% of all platelets are labelled). Shown are representative intravital microscopy images of platelet aggregates formed after five minutes. Blood flow was from top to bottom. N = 6 animals per group, bar is 250 µm.


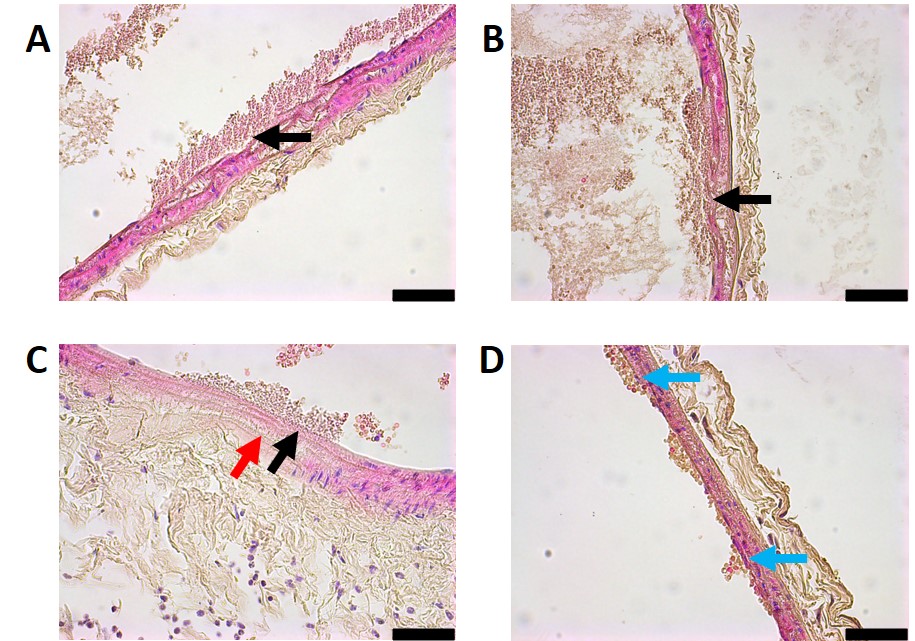


**Supplemental figure 3.** **Representative images of histological cross sections of right carotid arteries from mice 3-72 hours post ligation**. Haematoxylin and eosin staining of tissue sections of the right carotid artery: **A)** 3, **B)** 6, **C)** 24 and **D)** 72 hours post ligation. Black arrow indicates localised denudation of the endothelial layer, red arrow indicates necrosis in the vessel wall, and blue arrow indicates partial recovery of the endothelial layer by reappearance of nuclei underneath thrombi. Bar is 50 µm.


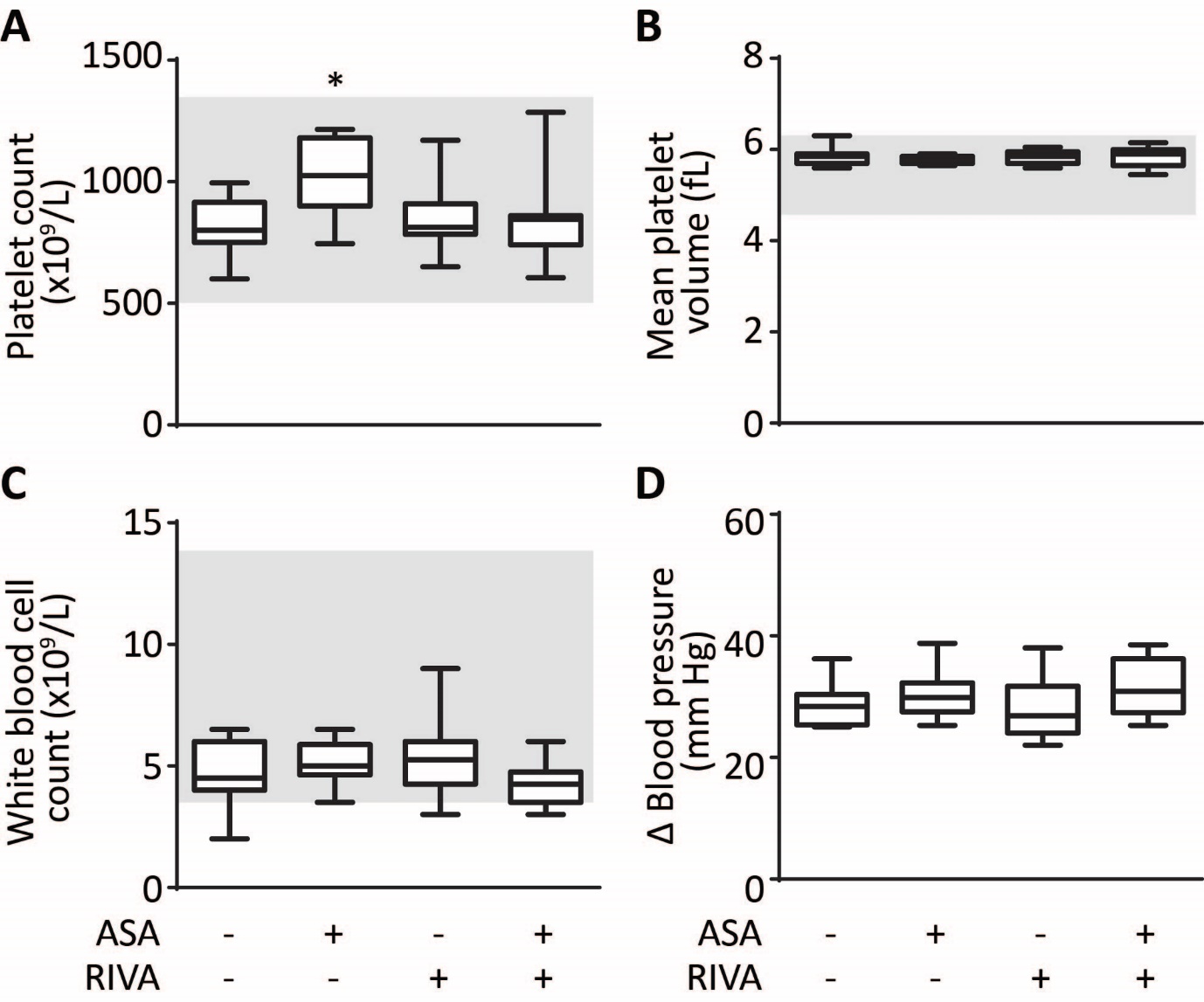


**Supplemental figure 4. Treatment with aspirin (ASA) and/or rivaroxaban (RIVA) did not affect whole blood cell count or blood pressure in C57BL/6 mice two weeks post‑ligation.** Shown are boxplots (min/max) of **A)** platelet count, **B)** mean platelet volume, **C)** white blood cell count, and **D)** difference in systolic and diastolic blood pressure. Reference values for C57BL/6 mice are given in grey. N = 11‑12 per group, *p < 0.05, one-way ANOVA.


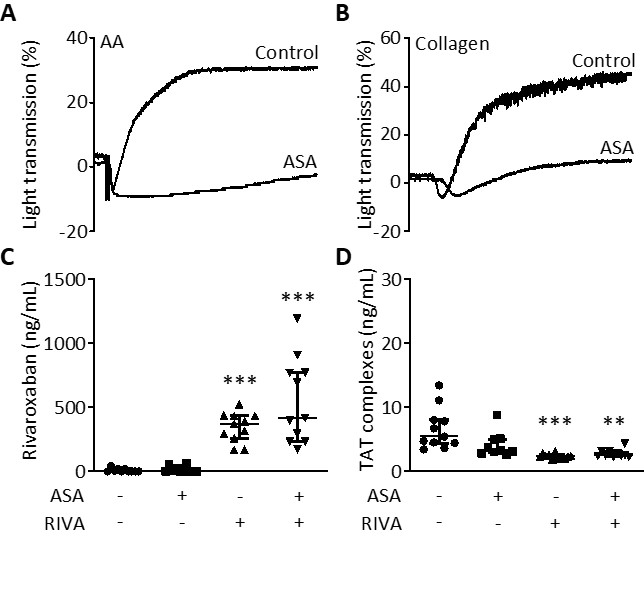


**Supplemental figure 5. Effect of mouse treatment with aspirin and/or rivaroxaban on platelet aggregation *in vitro* and on TAT complexes in plasma.** Light transmission aggregation was measured of washed platelets from mice receiving aspirin (ASA) or control animals. Representative aggregation traces, with a total duration of ten minutes, are given from three independent experiments. The platelets were stimulated with **A)** 12.5 µM arachidonic acid (AA) or **B)** 0.8 µg/mL Horm collagen, in buffer medium containing 2 mM CaCl_2_. Levels of **C)** rivaroxaban (RIVA) and of **D)** thrombin‑antithrombin (TAT) complexes were measured in platelet‑free plasma from mice treated with aspirin and/or rivaroxaban. Data are represented as scatterplots (median + IQR), n = 11‑12 animals per group, **p < 0.01, *** p < 0.001, Kruskal-Wallis test.

**
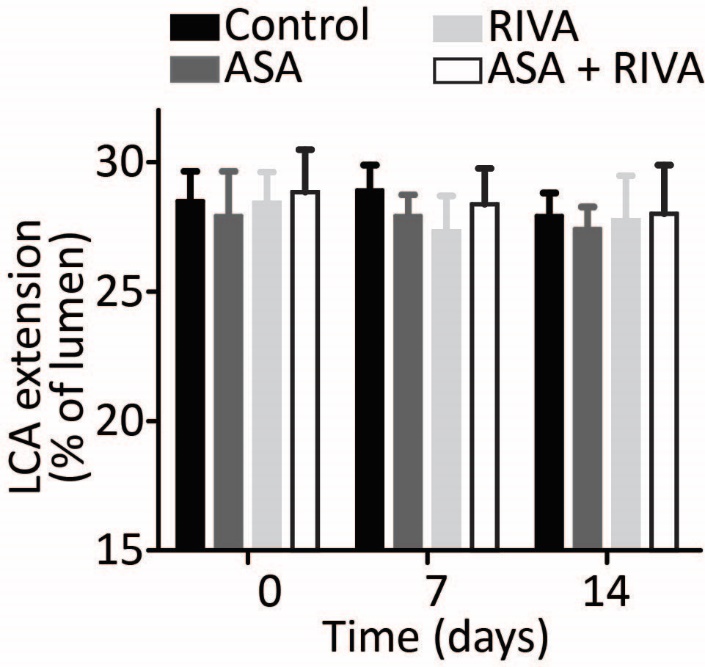
**

**Supplemental figure 6. Mouse treatment with aspirin and/or rivaroxaban does not affect vascular stiffening of the control left carotid artery.** Carotid vessel wall extension was measured in the four groups of mice using ultrasound (Vevo 2100) at baseline (day 0), 7 and 14 days after temporary ligation. Extension is expressed as % of lumen with quantification done in M‑mode. Left carotid artery (LCA) extension in control mice or mice treated with aspirin (ASA), rivaroxaban (RIVA) or the combination of both. Data are presented as mean ± SEM, n=11‑12 animals per group, **p < 0.01, two-way ANOVA.

**
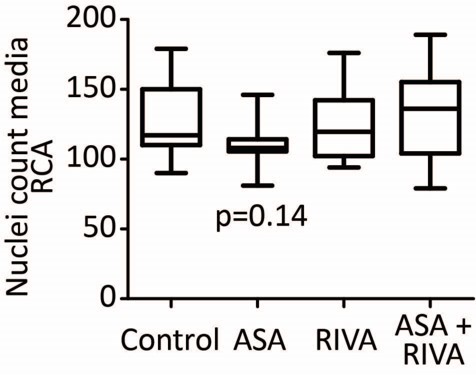
**

**Supplemental figure 7. Mouse treatment with aspirin and/or rivaroxaban does not affect nuclei count of the intima-media.** Haematoxylin and eosin staining of paraffin sections of the right carotid artery of mice treated with vehicle, aspirin (ASA), rivaroxaban (RIVA) or both, obtained 14 days after temporary ligation. Shown is a boxplot (min/max) with quantification of the nuclei count in the intima-media, n = 11‑12 animals per group, one-way ANOVA.


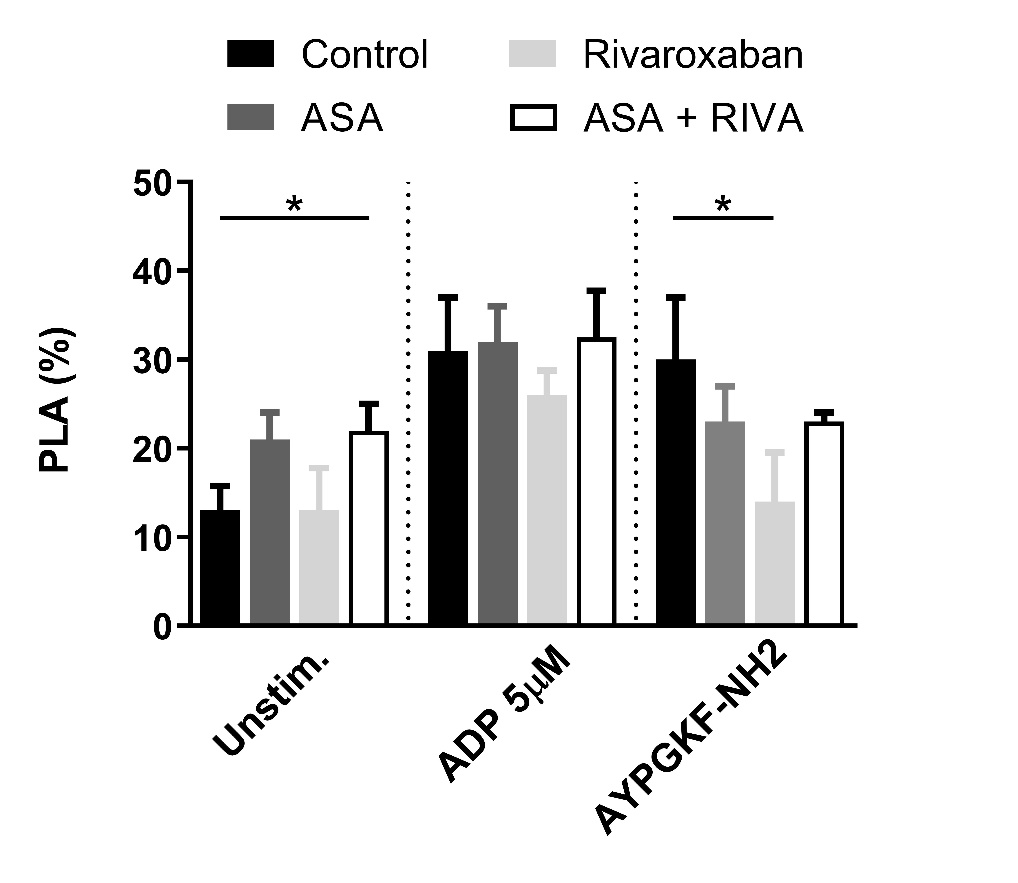


**Supplemental figure 8. Effect of therapy on percentage of leukocytes with bound platelets in unstimulated, 2‑MeSADP‑ or AYPGKF‑stimulated blood samples.** Blood was collected from mice at two weeks after temporary ligation of the carotid artery. During the post-ligation period, mice were treated with aspirin (ASA), rivaroxaban (RIVA), ASA + RIVA, or saline (control). Using flow cytometry, platelet-leukocyte aggregates (PLAs) were measured in whole blood by dual staining with APC αCD45 and DyLight 488-labelled αGPIbβ antibodies. Platelets were unstimulated, stimulated with 2-MeSADP (5 μM) or AYPGKF (30 μM). Shown are percentage of leukocytes with bound platelets. Data are represented as bar graphs (median + IQR), n = 7-12 animals per group, *p < 0.05, Kruskal-Wallis test.
